# Characterization and Modeling of Out-of-Plane Behavior of Fiber-Based Materials: Numerical Illustration of Wrinkle in Deep Drawing

**DOI:** 10.3390/ma17174177

**Published:** 2024-08-23

**Authors:** Cedric W. Sanjon, Yuchen Leng, Yi Yan, Peter Groche, Marek Hauptmann, Nicole Ludat, Jens-Peter Majschak

**Affiliations:** 1 Fraunhofer Institute for Process Engineering and Packaging IVV, 01189 Dresden, Germany; 2 TU Darmstadt Institute for Production Engineering and Forming Machines, 64287 Darmstadt, Germany; 3 TU Dresden Institute for Processing Machines and Processing Technology, 01062 Dresden, Germany

**Keywords:** paperboard, delamination, cohesive model, material characterization, material modeling, wrinkle, creases

## Abstract

The characterization and modeling of the out-of-plane behavior of fiber-based materials is essential for understanding their mechanical properties and improving their performance in various applications, especially in the forming process. Despite this, research on paper and paperboard has mainly focused on its in-plane behavior rather than its out-of-plane behavior. However, for accurate material characterization and modeling, it is critical to consider the out-of-plane behavior. In particular, delamination occurs during forming processes such as creasing, folding, and deep drawing. In this study, three material models for paperboard are presented: a single all-material continuum model and two composite models using different cohesion methods. The two composite models decouple in-plane and out-of-plane behavior and consist of continuum models describing the behavior of individual layers and cohesive interface models connecting the layers. Material characterization experiments are performed to derive the model parameters and verify the models. The models are validated using three-point bending and bulge tests and show good agreement. A case study is also conducted on the application of the three models in the simulation of a deep drawing process with respect to wrinkle formation. By comparing the simulation results of wrinkle formation in the deep drawing process, the composite models, especially the cohesive interface composite model, show greater accuracy in replicating the experimental results, indicating that a single continuum model can also be used to represent wrinkles.

## 1. Introduction

Fiber materials such as paper and paperboard play an indispensable role in numerous industrial applications such as printing, packaging, and construction. Paperboard is a fibrous material consisting primarily of wood pulp and can be produced as single-ply or multi-ply paperboard, depending on the end use. Multi-ply paperboard is more widely used because of the higher basis weights usually required for practical processing. Multi-ply paperboard is usually made by combining multiple layers of paper fiber webs using starch or adhesive materials [1]. Additionally, one- or two-sided coatings can be used to enhance the functionality of the paperboard, such as waterproofing and sealing.

Research on the out-of-plane behavior of paperboard is relatively limited compared to its in-plane behavior [2]. Due to the thinness of paperboard, it is common to consider these materials as two-dimensional planar structures, and the thickness direction is treated as purely elastic in modeling [3]. Moreover, during the 3D forming process, in-plane stresses typically dominate, while stresses in the thickness direction are often considered negligible. In addition, since paper is a planar, almost two-dimensional material, the properties in the thickness direction can be reduced to pure elasticity. However, in many engineering applications, the out-of-plane response of materials to applied loading is equally important. During various 2D forming processes, such as creasing and folding, the paperboard undergoes significant deformation and delamination between plies [4]. Minor delamination can also occur during three-dimensional forming processes, such as wrinkling during deep drawing. The failure processes are also quite difficult to tune in the models with paperboard. Therefore, it is critical to develop a model that captures both the deformation and breakage of the internal fiber network and the delamination between plies to better understand the material properties under different conditions.

**Figure 1:**
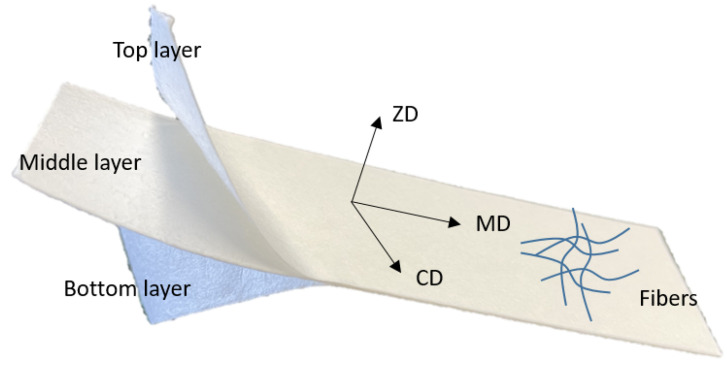
Illustration of delaminated paperboard structure.

Paper and paperboard exhibit significant anisotropic behavior due to the fiber orientation distribution during manufacturing. Figure 1 depicts the typical structure of multi-ply paperboard. In each layer, the majority of fibers align with the machine direction (MD), with a smaller portion aligning in the cross direction (CD). The thickness direction in which the fibers are least aligned is referred to as ZD. In other words, the fibers are predominantly aligned in the plane. Consequently, due to this fiber orientation, the mechanical properties of paper vary significantly between the MD-CD (in-plane) and ZD (out-of-plane) directions [2].

The material behavior of paperboard in the thickness direction, including interface damage, such as delamination, can be identified through a series of tests. The out-of-plane tensile test is typically performed by attaching the paper to metal plates with a double-sided adhesive tape. The specific material and positioning of the tools require particular attention, as the high transverse stiffness and absolute parallelism of the test tools contribute to the accuracy of the results [5]. In this experiment, the deformation of the fiber network and interlayer delamination occur simultaneously, making it impossible to directly obtain the out-of-plane behavior, such as the elastic modulus. Consequently, out-of-plane cyclic compression tests were conducted by Stenberg [6] to characterize the elastic–plastic behavior in the ZD.

Several methods are available for testing shear behavior, including the rigid support test with an Arcan device [7], the rigid block method [8], and the Iosipescu method [9]. However, the penetration of materials used for adhesion could affect the results. To overcome these limitations, the double-notch shear test, V-notched shear test, and strip shear test were proposed by Nygårds [10]. In these tests, the sample was notched, and shear damage occurred between them during loading, making adhesion unnecessary. Nevertheless, this also means that sample preparation could be challenging due to the need to make notches prior to experimentation. Specific tests were also employed to assess delamination behavior and to measure delamination resistance, such as the Scott bond test [11].

For numerical stability, the through-thickness direction (ZD) is generally treated separately in modeling, assuming an out-of-plane Poisson’s ratio of zero in the elastic region [12]. Stenberg et al. [13] developed an elastic–plastic model for the combination of low compressive loads and out-of-plane shear under the assumption of small-strain orthotropic linear elasticity and a quadratic yield function with an associative flow rule. This model did not account for plastic deformation in tension and delamination behavior. Hallbäck et al. [14] modeled paperboard as a four-layer structure with a softening interface model linking the paperboard plies. The interface behavior was described by an orthotropic elastic–plastic cohesive law, which relates the interface traction to the opening and sliding of the interface, as referred by Xia [15]. For computational efficiency, interface models simulated the interaction between the top and upper middle layers, as well as between the middle layers of the board. The bottom layer was assumed to be bonded to the lower middle layer. Nygårds [16] presented a material model consisting of a continuum and an interface model for out-of-plane behavior. The continuum model is elastic–plastic in both shear and compressive directions but elastic only in the tensile direction. Two different yield surfaces initiate plastic deformation: one for compression and the other for a combination of shear and normal stresses. Interface segregation was determined using criteria that combined normal and shear stress tensions. The developed model was used to simulate combined compression and shear stresses. Borgqvist et al. [17] modeled the out-of-plane properties based on the in-plane yield surface of Xia et al. [15] for large plastic strains. The free energy model first produces an uncoupled in-plane and out-of-plane behavior, allowing for the modeling of the large differences in in-plane and out-of-plane elastic parameters. Plasticity was modeled with a single distortional hardening yield surface, considering only ZD compression. Li et al. [18] proposed an out-of-plane elastic–plastic model with a quadratic yield locus based on experimental observations in tension, compression, and combined compression–shear tests. The model accounted for both material compression and inner friction effects, and it accurately captured the highly anisotropic, elastic-plastic behavior.

There have also been some studies on modeling the interface behavior of paperboard at the fiber level. Simonetto et al. [19] developed a method for modeling failure in paper-based sandwich materials using a combination of fiber-based and cohesive numerical modeling approaches. To model the interface conditions between the various layers, a bilinear traction-separation cohesive model dependent on the fracture mode, i.e., normal (mode I), tangential (mode II), or mixed, was implemented. The results demonstrated the effectiveness of the model in accurately predicting fiber behavior during deformation. The cohesive modeling strategy can provide a theoretical basis for describing the nonlinear behavior and damage mechanisms of materials. In addition to paperboard materials, the delamination phenomenon is also common in laminated composites, and the proposed cohesive modeling approach is useful. In [20], it is proposed that the buckling response is based on certain cases of the distribution of material properties between phases due to the mixing of the two phases during the layer-by-layer curing process. The presence of inhomogeneous phases between the two main constituents in a hyperelastic layered composite significantly alters its buckling behavior, affecting the onset of instability as well as the development of buckling modes. Furthermore, the buckling characteristics appear to depend not only on the thickness and effective shear modulus of the interphase layers, but also on the distribution of the shear modulus across their thickness. In a recent study [21], an innovative theoretical framework based on nonlinear homogenization was proposed to describe the damage behavior of periodically reinforced hyperelastic composites. This framework is designed to account for damage induced by reinforcement/matrix debonding, as well as by the interaction between the contact mechanism and microscopic instabilities. The framework incorporates debonding and unilateral contact between different phases in the context of continuum mechanics. This is achieved through the use of an extended cohesion/contact model, which features a special nonlinear interfacial constitutive law and an accurate contact formulation.

In earlier studies, 3D forming processes of paperboard, such as deep drawing, press forming, and hydroforming, were simulated using different numerical models and approaches. Wallmeier et al. [3] found that tools with a smooth surface roughness significantly reduced the probability of failure during deep drawing by examining the influences of the blank holder force, die temperature, and paperboard thickness. Awais et al. [22] numerically examined the impact of the number of creases on the formability of the paperboard, where the creases were modeled using hinge connector elements rather than explicitly included in the geometry. Their results showed that increasing the number of creases helped to reduce the distortion at tray edges. Linvill et al. [23] further explored the use of hinge connector elements for modeling creases. This approach allows for spontaneous wrinkling, meaning that the number and location of the wrinkles need not be predetermined. The results of the deep drawing simulations indicated that the number of creases formed was comparable to the experimental observations. However, these methods were unable to evaluate the effect of the wrinkles on the frictional interaction between the paperboard and the forming device because the wrinkles were not physically resolved. Lindberg and Kulachenko [24] employed an orthotropic material model with isotropic hardening based on Hill plasticity, accounting for differences in tension and compression regarding yielding and damage. They introduced a detailed geometric resolution of creases using more elements than previous studies, improving precision in studying crease effects on frictional interaction. They used implicit time integration to enhance the simulation accuracy and analyzed the frictional conditions required for the desired shape. However, the wrinkle distribution is still not uniform due to process parameters and material inhomogeneities. Consequently, the implementation of wrinkles in the geometry was constrained and did not encompass the impact of process parameters and material structure.

This work presents and compares two different cohesive modeling methods; they were applied to the simulation of wrinkles in the deep drawing process. Various experiments were conducted to characterize the material behavior, both in-plane and out-of-plane. The developed material models describe the in-plane deformation of the inner fiber network and the out-of-plane delamination between layers. To validate the model, a single continuum model, a multilayer composite model with cohesive elements, and a multilayer composite model with cohesive interactions were compared using 2D and 3D bulge tests. Finally, a simulation case study of the deep drawing process was conducted to compare wrinkle formation using the three material models.

## 2. Materials and Methods

### 2.1. Materials

Paperboard, used in this work, is a bleached three-layer virgin-fiber board with chemithermomechanical pulp (CTMP) in the middle layer and sulfate pulp in the outer layers, specifically designed for folded and pressed trays. The grammage of the paperboard is 310 g/mm^2^ and the thickness is 420 μm. Here, each of the three layers is considered as a continuum model, while the adhesive between the layers is modeled with a cohesive zone model.

### 2.2. Material Characterization Experiments

#### 2.2.1. In-Plane Uniaxial Tensile Test

Whether for the whole paperboard or the individual layers, uniaxial tensile tests in 3 directions, namely MD, 45°, and CD, are required to determine the in-plane mechanical property. The material testing machine Zwick Z100 and GOM Aramis 5M were utilized for force and deformation measurements during the tensile test. The tensile specimen had a geometry of 120 × 30 mm^2^ with a clamping length of 90 mm, and the tensile speed was 20 mm/min. For the multilayer modeling, the entire sheet of paperboard was delicately torn into individual layers before testing. It was imperative to preserve the integrity of the paperboard fibers in the specimens, as they would remain in use. Thus, the use of adhesive tapes, liquid glues, or other aids was avoided, and the paperboard was not moistened or heated. The specimens were carefully layered by hand into three layers, and several attempts and measurements of the thickness of the individual layers ensured that the layering always occurred between the production layers without offsetting. If it tore at fracture/shear surfaces that are not related to the formation structure, it would lead to modeling inaccuracies.

#### 2.2.2. In-Plane Shear Test

**Figure 2:**
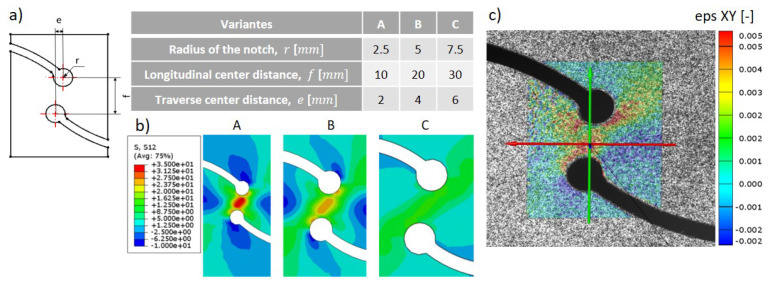
(**a**) Geometric parameters of in-plane shear sample. (**b**) Shear stress (in MPa) cloud charts in the simulation. (**c**) Shear stress distribution using GOM Aramis (Geometry A).

Furthermore, an in-plane shear test with a specially designed geometry was conducted to validate the parameters derived from the equation in the literature for the material model (Equation (3)). The shear specimen had exterior dimensions of 120×50 mm^2^ and a clamping length of 80 mm. The interior shape of the specimen was designed after the template by Kolupaev [25] to achieve a pure shear field in the central region. Compared to the conventional slotted shear specimen, the spatial geometry of the new optimized specimen allowed for a more homogeneous shear field and reduced stress concentrations at the top of the slots. The stress distribution varied depending on the shape of the specimen. The influential parameters were the notch radius *r*, the longitudinal distance *f*, and the traverse center distance *e* (ref. Figure 2a). Simulations under boundary conditioning with a fixed lower end and a uniformly displaced upper end were conducted to compare three different combinations of the shape parameters, as shown in Figure 2b. By analyzing shear stress cloud charts obtained from various specimens, it can be seen that geometry A has the most uniform shear stress distribution. It is important to note that when the shear zone of the specimen is smaller, it can make the preparation of the specimen more challenging and the accurate measurement of the resulting smaller tensile force more difficult. Therefore, geometry A was selected as the geometry for the in-plane shear test specimen. Because of the limited shear stress region, the in-plane shear test does not analyze the deformation of the entire specimen. Instead, it employs high-precision photogrammetry and a smaller measurement volume of 50×45×25 mm^3^. Due to the limited deformation observed in the shear test, some measurement noise was unavoidable, but it still showed a similar distribution to the simulation as shown in Figure 2c).

#### 2.2.3. Out-of-Plane Compression Test

Compared to typical in-plane experiments, determining the mechanical properties in the out-of-plane direction is challenging due to the material’s thinness. To establish the elastic modulus in the thickness direction without delamination, out-of-plane compression tests were first conducted on both the entire paperboard and the individual layers. The experiments were conducted using Zwick Z100 (Ulm, Germany), equipped with a non-contact high-resolution video extensometer. Two markers are attached to the upper and lower plates of the compression testing equipment to precisely measure small deformations that occurred during a test. This is performed to avoid any errors caused by machine stiffness or tool deformation. The sample geometry was a square of 20×20 mm^2^ and the compression rate was 1 mm/min.

#### 2.2.4. Out-of-Plane Tensile Test

**Figure 3:**
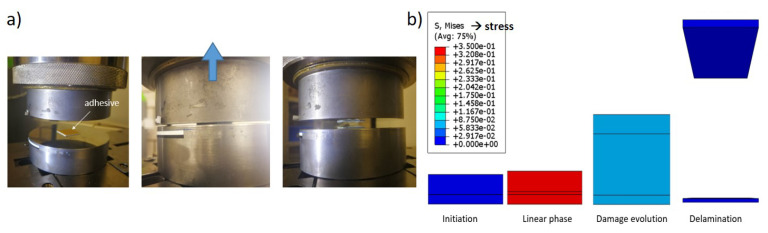
(**a**) Schematic illustration of the out-of-plane tensile test. (**b**) Simulation model of the experiment.

The out-of-plane tensile test exhibits a more intricate set of phenomena than the compression test, including the deformation of individual paperboard fiber networks and the occurrence of delamination along the z-direction displacement. When dealing with multi-ply paperboard, the out-of-plane tension behavior is dominated by delamination occurring between layers. Moreover, for a three-ply paperboard, i.e., a paperboard with two layered interfaces, the weaker interface tends to be the site of delamination for the entire material. Therefore, parts of the material containing two layers and an interface were tested instead of the entire material for this research. The tensile test was conducted similarly to the out-of-plane compression test, using the Zwick Z100 and a video extensometer. The compression tool plates and specimen geometry were also consistent between both tests. The tensile speed was 10 mm/min. In the out-of-plane tensile test, it was necessary to firmly bond the specimen to both the top and bottom plates using a tesafix^®^ 4965 (tesa, Hambuger, Germany) double-sided adhesive tape, as shown in Figure 3. This prevents any possible movement or displacement of the specimen from the tooling. For a three-ply paperboard, the interlayer properties were measured individually by tearing off either the first or third layer first. This provided information on the delamination behavior between the second and third layers or the first and second layers. Furthermore, the deformation of the fiber network itself was insignificant in this experiment. Figure 3b shows the numerical model of the out-of-plane tensile test and the simulation process.

#### 2.2.5. Out-of-Plane Shear Test

**Figure 4:**
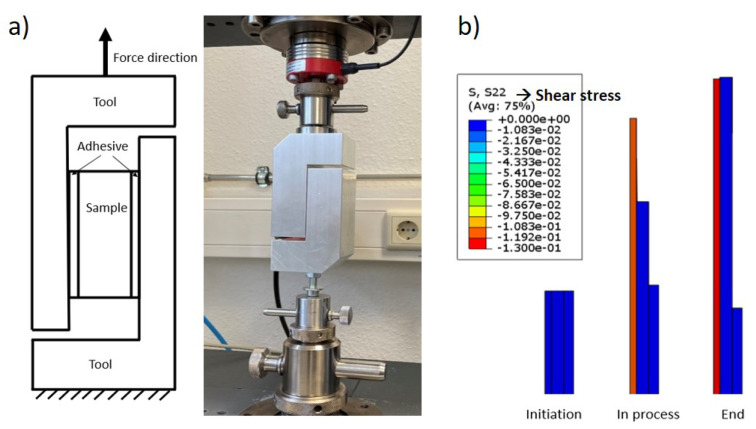
(**a**) Schematic illustration of the out-of-plane shear test. (**b**) Simulation model of the experiment.

To determine parameters, such as shear modulus and shear stiffness, tools have been designed for out-of-plane shear testing (ref. Figure 4). The tools were mounted on Zwick Z005 with a 1 kN load cell. Each face of the specimen was glued between two rigid blocks using a tesafix^®^ 4965 double-sided adhesive tape, and the tensile tester recorded the force and displacement. The specimen was tested in both directions, along and perpendicular to the tensile direction, with a geometry of 20×20 mm^2^. Additionally, the adhesive force of the tape alone was also tested without the paperboard specimens to eliminate the part of the experimental results contributed by the tape. Due to the anisotropy of the paperboard, the shear properties in the MD-ZD and CD-ZD directions were measured separately. However, for this material, no delamination was observed until the specimen was detached, which indicates that the adhesive force is less than the shear delamination force. Therefore, it is assumed that delamination occurs at the end of the experiment and that the shear characteristics are the same in the MD-ZD and CD-ZD directions. Figure 4b shows the numerical model of the out-of-plane shear test and the simulation process.

### 2.3. Material Modeling

**Figure 5:**
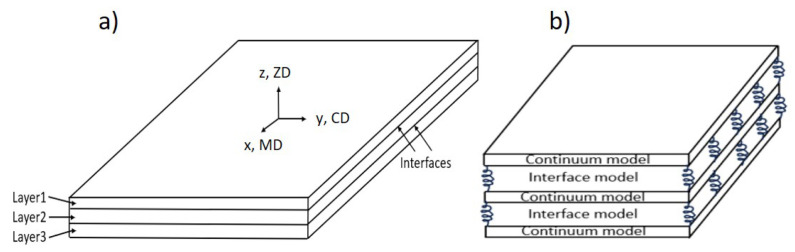
Composite material modeling with consideration of delamination behavior: (**a**) model with cohesive interaction, (**b**) model with interface part with different properties.

Paperboard can be modeled as a single continuum model. Moreover, considering the delamination behavior of the multi-ply paperboard, the material model can be presented by the multilayer model, which consists of the continuum model of every layer and the interface model connecting the layers, as schematically illustrated in Figure 5. Three different material models have been developed, two of which are composite models considering delamination behavior: one with the interface as a part with material properties, another with the interface as a cohesive interaction, and finally, a simple single solid continuum model for comparison. The in-plane fracture generally appears later in the CD than in the MD for paperboard under the uniaxial tension. Hence, in this work, CD is chosen as the first principal direction in numerical simulation.

#### 2.3.1. In-Plane Continuum Modeling and Validation

Regarding its in-plane behavior, paperboard is an anisotropic material that can be approximated as being orthotropic. It displays varying behavior in response to tension and compression. Therefore, a stress state utilizing Hill’s yield criterion [26] and multilinear isotropic hardening law is employed.

The orthotropic elastic material model is shown as follows:

(1) $$\left[\begin{array}{c} \epsilon_{xx}\\ \epsilon_{yy}\\ \epsilon_{zz}\\ \epsilon_{yz}\\ \epsilon_{zx}\\ \epsilon_{xy} \end{array}\right]=\left[\begin{array}{cccccc} \frac{1}{E_{x}} & -\frac{\nu_{yx}}{E_{y}} & -\frac{\nu_{zx}}{E_{z}} & 0 & 0 & 0\\ -\frac{\nu_{xy}}{E_{x}} & \frac{1}{E_{y}} & -\frac{\nu_{zy}}{E_{z}} & 0 & 0 & 0\\ -\frac{\nu_{xz}}{E_{x}} & -\frac{\nu_{yz}}{E_{y}} & \frac{1}{E_{z}} & 0 & 0 & 0\\ 0 & 0 & 0 & \frac{1}{2G_{yz}} & 0 & 0\\ 0 & 0 & 0 & 0 & \frac{1}{2G_{zx}} & 0\\ 0 & 0 & 0 & 0 & 0 & \frac{1}{2G_{xy}} \end{array}\right]\left[\begin{array}{c} \sigma_{xx}\\ \sigma_{yy}\\ \sigma_{zz}\\ \sigma_{yz}\\ \sigma_{zx}\\ \sigma_{xy} \end{array}\right]$$

As previously stated, the decoupling between in-plane and out-of-plane behaviors assumes the Poisson’s ratio in the out-of-plane directions to be 0.

(2) $$\nu_{xz}=\nu_{zx}=\nu_{yz}=\nu_{zy}=0,$$

The in-plane shear modulus $G_{xy}$ is given by the empirical expression [27]:

(3) $$G_{xy}=\frac{1}{\frac{4}{E_{45}}-\frac{1}{E_{x}}+2\frac{\nu_{xy}}{E_{x}}-\frac{1}{E_{y}}}$$

and

(4) $$G_{xz}=G_{yz}=G_{xy},$$

And Poisson’s ratio $\nu_{xy}$ is measured from the tensile test conducted with DIC technology, which provides a more accurate result. This value also corresponds with the following equation [28]:

(5) $$\nu_{xy}=0.293\sqrt{\frac{E_{x}}{E_{y}}}$$

To determine $E_{x}$, $E_{y}$, $E_{45}$, and $E_{z}$ for Young’s modulus and the initial yield stress $\sigma_{xx}^{0}$, $\sigma_{yy}^{0}$, $\sigma_{45}^{0}$, and $\sigma_{zz}^{0}$, linear regression is used, which allows for the determination of linearity at the beginning of the stress–strain curve. An alternative approach would be to employ reverse engineering for the determination of the parameters. The plastic behavior of the material is assumed to follow Hill’s yield criterion [26], as expressed in terms of the ratio $R_{ij}$ of the yield stress in direction ij concerning a reference direction. It is an extension of the von Mieses yield criterion accounting for the anisotropic yield of the material, expressed as follows:

(6) $$\sigma^{y}=\sqrt{H{\left(\sigma_{xx}-\sigma_{yy}\right)}^{2}+F{\left(\sigma_{yy}-\sigma_{zz}\right)}^{2}+G{\left(\sigma_{zz}-\sigma_{xx}\right)}^{2}+2N\sigma_{xy}^{2}+2L\sigma_{yz}^{2}+2M\sigma_{zx}^{2}}$$

where $\sigma_{ij}$ (i = x, y, z and j = x, y, z) is the stress component and H, F, G, L, M, N are anisotropic material constants, which can be defined as follows

(7) $$\begin{array}{c} F=\frac{1}{2}\left(\frac{1}{R_{yy}^{2}}+\frac{1}{R_{zz}^{2}}-\frac{1}{R_{xx}^{2}}\right)\\ G=\frac{1}{2}\left(\frac{1}{R_{zz}^{2}}+\frac{1}{R_{xx}^{2}}-\frac{1}{R_{yy}^{2}}\right)\\ H=\frac{1}{2}\left(\frac{1}{R_{xx}^{2}}+\frac{1}{R_{yy}^{2}}-\frac{1}{R_{zz}^{2}}\right)\\ L=\frac{3}{2}\frac{1}{R_{yz}^{2}}\\ M=\frac{3}{2}\frac{1}{R_{xz}^{2}}\\ N=\frac{3}{2}\frac{1}{R_{xy}^{2}} \end{array}$$

with

(8) $$\begin{array}{c} R_{xx}=\frac{\sigma_{xx}^{y}}{\sigma^{y}};R_{yy}=\frac{\sigma_{yy}^{y}}{\sigma^{y}};R_{zz}=\frac{\sigma_{zz}^{y}}{\sigma^{y}};\\ R_{xy}=\sqrt{3}\frac{\sigma_{xy}^{y}}{\sigma^{y}};R_{yz}=\sqrt{3}\frac{\sigma_{yz}^{y}}{\sigma^{y}};R_{zx}=\sqrt{3}\frac{\sigma_{zx}^{y}}{\sigma^{y}} \end{array}$$

and $\sigma^{y}$ is the yield stress that can, in general, evolve as a function of some material internal variables.

(9) $$\sigma^{y}=a\left(1-e^{-b{\hat{\epsilon }}_{pl}}\right)+c{\hat{\epsilon }}_{pl}^{\frac{1}{d}}+\sigma_{0}$$

where a, b, c, and d are plastic parameter constants, $\sigma_{0}$ is the initial yield stress, and ${\hat{\epsilon }}_{pl}$ is the equivalent plastic strain. It should be noted that Equation (9) is fitted to the response in the y-direction since $R_{yy}$ = 1, the in-plane shear parameter is determined with the behavior in a 45° tensile direction, and the plastic behavior in the ZD is assumed to have the same value as the y-direction $R_{zz}$ = 1.

The material property constants were derived from uniaxial tensile tests in three directions and out-of-plane compression test and fitted to the above plastic flow equation, as shown in Table 1.

**Figure 6:**
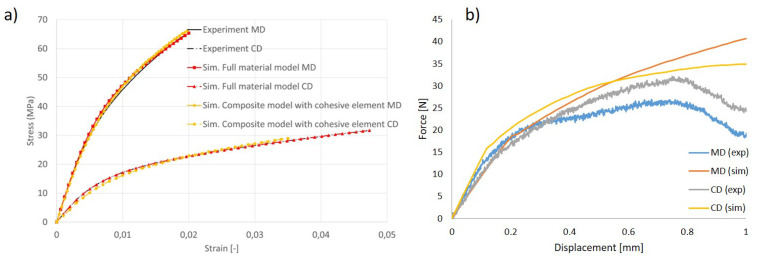
(**a**) Validation of continuum model and composite model using in-plane tensile tests in 3 directions. (**b**) Validation of continuum model using in-plane shear test.

The comparison of the experimental and simulation results is illustrated in Figure 6a, which demonstrates a high degree of agreement. Both the full material continuum model and the composite model including cohesive elements are accurate for the numerical reproduction of tensile tests. As for the in-plane shear test, as demonstrated in Figure 6b, the relationship between shear stress and the simulation results is consistently observed. The simulation results are also validated by experiments, which, in turn, verify the empirical formula (Equation (3)). It also shows that the results of the self-designed in-plane shear experiments are reliable, and that similar geometries can be used if one is interested only in the shear properties or related parameters such as shear modulus and shear angle.

#### 2.3.2. Out-of-Plane Interface Modeling

**Figure 7:**
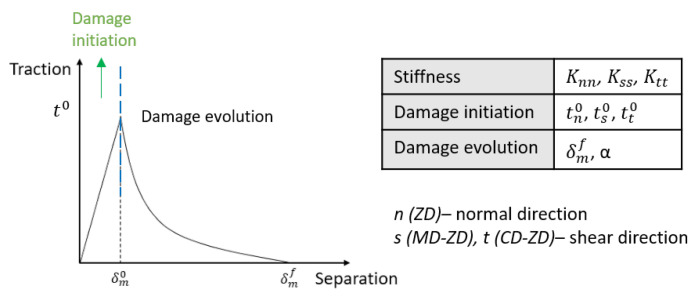
Interface model: damage initiation criterion and relevant parameters.

The interface model with a traction-separation description, shown in Figure 7, consisting of linear damage initiation and exponential damage evolution, is used. $K_{nn}$ represents the stiffness in ZD, and $K_{ss}$, $K_{tt}$ are the shear stiffnesses in MD and CD, while $t_{i}$ and $\delta_{i}$ denote the traction and displacement. The parameter α, which represents the exponential law, is validated through the simulation process. The initial behavior of the cohesive element is linear, as follows:

(10) t=K·δ

Damage appears and degradation begins once the damage initiation criterion is reached. The maximum nominal stress criterion assumes that the damage appears when any of the stresses reach its maximum nominal stress. The criterion is expressed as

(11) $$max\left\{\frac{\langle t_{n}\rangle }{t_{n}^{0}},\frac{t_{s}}{t_{s}^{0}},\frac{t_{t}}{t_{t}^{0}}\right\}=1$$

where $t_{i}^{0}$ denotes the maximum nominal stress. The exponential softening law describes the degradation rate of the material stiffness. The material degradation is introduced by a scalar damage variable *D*, which increases from 0 to 1 with the damage evolution procedure. The change of stress components of the traction–separation model with the degradation is expressed as

(12) $$t_{n}=\left\{\begin{array}{cc} \left(1-D\right)t_{n}^{0}, & t_{n}^{0}\geq 0\\ t_{n}^{0}, & otherwise \end{array}\right.$$

For exponential damage evolution, the scalar damage variable *D* is defined as [29]

(13) $$D=1-\frac{\delta_{m}^{0}}{\delta_{m}^{max}}\left[1-\frac{1-e^{-\alpha \left(\frac{\delta_{m}^{max}-\delta_{m}^{0}}{\delta_{m}^{f}-\delta_{m}^{0}}\right)}}{1-e^{-\alpha }}\right]$$

where α is a nondimensional material parameter, which defines the material degradation rate. $\delta_{m}$ is the effective displacement, describing the damage evolution under a combination of normal and shear separations across the interface, defined as

(14) $$\delta_{m}=\sqrt{{\langle \delta_{n}\rangle }^{2}+\delta_{s}^{2}+\delta_{t}^{2}}$$

In Equation (13), $\delta_{m}^{0}$, $\delta_{m}^{f}$, and $\delta_{m}^{max}$ denote the relative effective displacement at damage initiation, effective displacement till complete failure, and maximum effective displacement attained during the loading history, respectively.

Two common methods for simulating cohesive behavior in finite element software are the insertion of cohesive materials and the definition of cohesive contact interactions. The former method entails the insertion of cohesive seams on adjacent surfaces after the meshing of the continuum model, thereby generating a zero-thickness interface model. Subsequently, the cohesive material model, which is specified in terms of elasticity and material damage initiation criteria, is then assigned to the cohesive seam elements. There are various types of damage, one of which is traction separation damage, which is described in the previous section. An alternative approach is to define cohesive contact interactions between two surfaces adhering in contact. Similarly to the cohesive material model, the interaction properties comprise cohesive behavior and damage evolution. In order to restrict the cohesive behavior to the surfaces in contact at the outset of the step, it is possible to select secondary nodes that are initially in contact.

The interface model comprises three stiffness values in the normal direction and two in the shear direction. These values can be determined from the ZD tensile and shear test. Damage initiation is defined by the maximum nominal stress criterion. Damage occurs when the nominal stress in any direction exceeds the stress limit, which can be determined from experimental results. Assuming a pure linear response of cohesive elements in the simulation, the maximum nominal stress values are slightly higher than the experimental values. Results of the out-of-plane tensile test show that the post-peak damage evolution follows an exponential decay. In FE simulation, this evolution can be defined by two parameters (refer to Figure 7), maximum effective displacement $\delta_{m}^{max}$, and exponential law parameter α, which can be calculated from the out-of-plane tensile test and verified by simulation results, as shown in Table 2.

**Figure 8:**
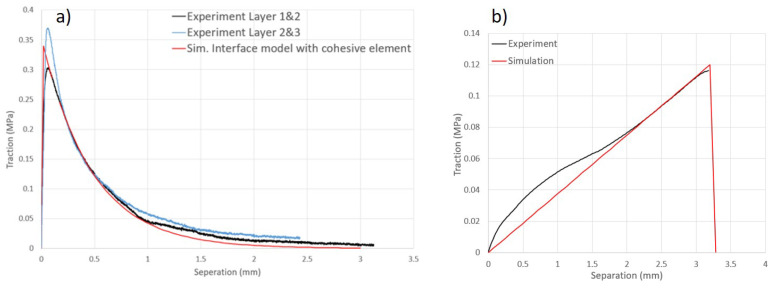
(**a**) Validation of cohesive model using out-of-plane tensile tests. (**b**) Validation of cohesive model using out-of-plane shear test.

The interface model effectively characterizes the initial phase of the softening evolution in out-of-plane tension by comparing numerical simulations to experimental results, as shown in Figure 8. The simulation results in Figure 8a are in good agreement with the experimental results and also demonstrate the feasibility of the material model and the correctness of the material parameters. The cohesive zone model characterizes well the initial phase of the softening evolution in out-of-plane tension. However, in the flat segment, due to the residual stress, the tensile force remains at a certain value with increasing separation distance instead of decreasing steadily to zero, which is different from the simulation results. The traction–delamination force relationship of the out-of-plane shear test is plotted in Figure 8b, and although the shear strength of the interface is weaker than the real material properties, it can still be described more accurately in the simulation. For shear behavior in the thickness direction, the traction–separation relationship is assumed to be linear before researching the maximum traction in the cohesive zone model.

However, in the flat segment, the traction maintains a certain value with increasing separation displacement due to residual stress, instead of dropping steadily to zero as seen in the simulation results. For the out-of-plane shear behavior, a linear traction–separation relationship is assumed until the maximum traction in the interface model is reached.

**Figure 9:**
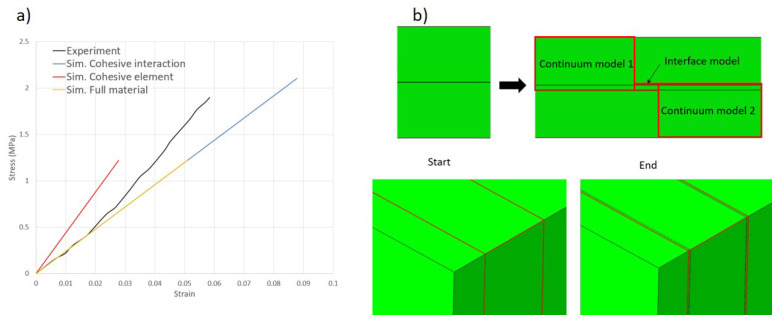
(**a**) Comparison of three material models for out-of-plane compression test; (**b**) Penetration of cohesive elements into interface model.

When comparing the two mentioned interface modeling methods, the simulated results for both in-plane tensile tests, out-of-plane tensile tests, and shear tests are nearly identical. However, a difference is noticeable in the out-of-plane compression tests. Figure 9 shows that the results of the single continuum model and multilayer composite model with cohesive interaction are consistent and slightly conservative compared to the experimental results. However, there is a significant difference in the results of the multilayer composite model with cohesive elements. Note that in the simulation software, the cohesive model assumes no damage under pure compression. Although the behavior of the interface model in the out-of-plane test is purely linear, it should not affect ZD compression due to the zero thickness of the model. However, during the out-of-plane compression simulation, cohesive elements penetrated into the continuum model, resulting in a lower continuum model strain than the logarithmic strain of the entire material, as shown in Figure 9b.

### 2.4. Validation Experiments and Deep Drawing Process

**Figure 10:**
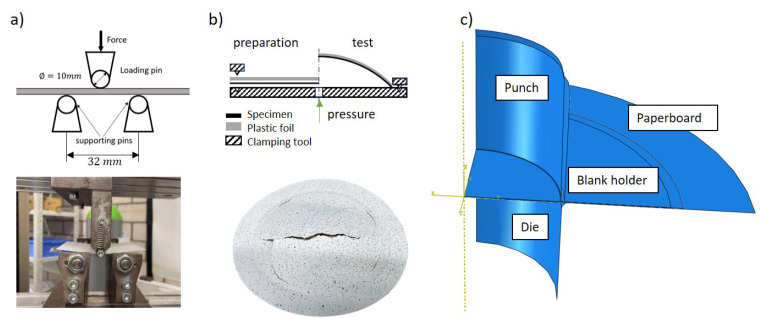
(**a**) Experimental setup of the three-point bending test. (**b**) Experimental setup and samples of the bulge test. (**c**) Geometric model of deep drawing simulation.

The three-point bending test and bulge test were conducted to validate and compare the three proposed models. Firstly, a three-point bending test (Figure 10a) was conducted to validate and compare the three composite material models. This test was conducted on a universal testing machine with a three-point bend fixture. The pneumatic bulge test is a multiaxial test for characterizing the material behavior, whose setup is shown in Figure 10b. The strain distribution on the specimen during the testing is measured with an optical DIC (digital image correlation) system (GOM Aramis). Additionally, wrinkling is a common defect in deep drawing parts, particularly with ordinary paperboard and a high drawing depth, that usually cannot be avoided [3]. It is caused by the build-up of excess material, usually in the flange area because of excessive compression stress, resulting in localized buckling of the part. A blank holder force is usually considered to be the most important factor influencing the formation of wrinkles. Abaqus/Explicit is utilized to simulate the deep drawing process. The workpiece, which is a paperboard, is deformable and has mesh element type C3D8R, while the work tools are rigid and have mesh element type C3D4. All geometric parameters of the experimental setup and boundary conditions used in the simulation of the deep drawing process are shown in Figure 10c and Table 3.

**Figure 11:**
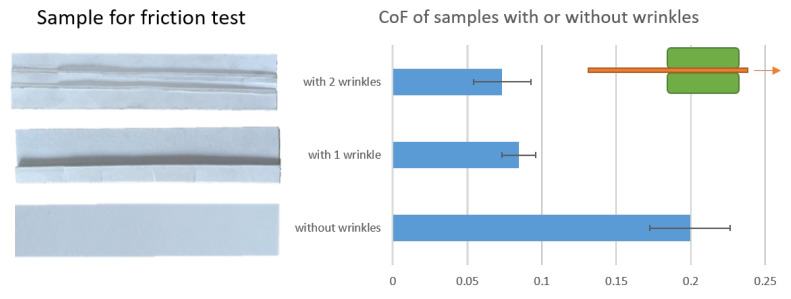
Comparison of friction coefficients of samples with two, one, and no wrinkles.

Among these, the friction coefficient was measured using a strip drawing test rig for fibrous materials and plastics at PtU, TU Darmstadt. In previous studies [30], the value of the friction coefficient was typically assumed to be large for simulation. However, this assumption is not appropriate for the deep drawing process, which is known to produce more wrinkles. Consequently, friction experiments were conducted on paper strips exhibiting two, one, and no wrinkles. As illustrated in Figure 11, the generation of wrinkles markedly diminishes the coefficient of friction.

## 3. Results

### 3.1. Validation Results of Bending and Bulge Tests

**Figure 12:**
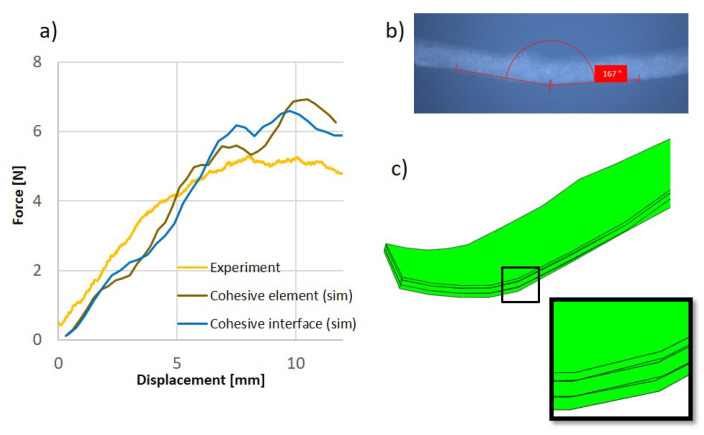
(**a**) Comparison of the experimental and numerical results of the bending test. (**b**) Microscopic Observation. (**c**) Delamination in the simulation.

Figure 12a compares the experimental and simulation results for the two multilayer composite models, showing close agreement despite measurement inaccuracies due to the resolution of the force sensors. The specimen was also photographed using the Zeiss Smartzoom 5 (Oberkochen, Germany), as shown in Figure 12b. The photograph reveals protrusions inside the cross-section at the center of the bend and a small delamination in the protrusion. In addition to the consistency demonstrated by the force–displacement curves in the experiment and simulation, delamination phenomena similar to those in the experiment can also be visualized in the simulation with a consideration of friction (see Figure 12c).

**Figure 13:**
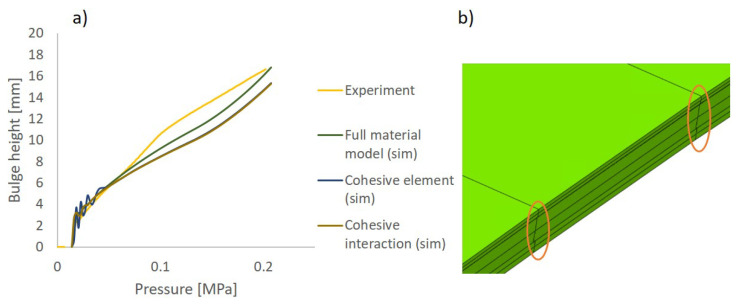
(**a**) Comparison of the experimental and numerical results using 3 different material models of the bulge test. (**b**) Slippage in simulation.

Figure 13a shows the relationship between the bulge height and pressure in the bulge test and simulation. It can be seen that among the three material models, both the multilayer composite model and the single continuum model are closer to the experimental results without significant differences. This is due to the fact that in the bulge test, the force on the material is mainly in-plane, and there is almost no deformation except at the blank holder out-of-plane. As a result, no delamination of the specimens is observed in the experiments. However, in numerical modeling, a small sliding of the interlayer elements can be observed in the central part of the specimen (Figure 13b) but not enough to form delamination. This demonstrates that multilayer composite models are no less capable of representing in-plane behavior than single continuum models.

### 3.2. Simulation of Wrinkles in the Deep Drawing Process

**Figure 14:**
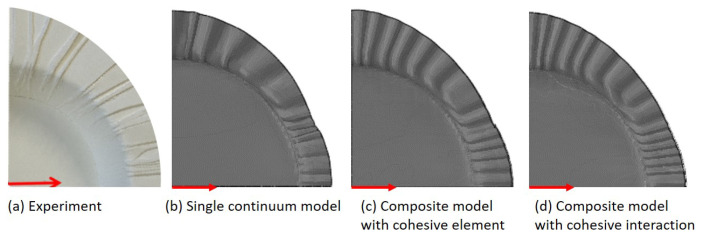
Comparison of experimental and simulation results of three material models (top view, direction of red arrow: MD).

**Figure 15:**
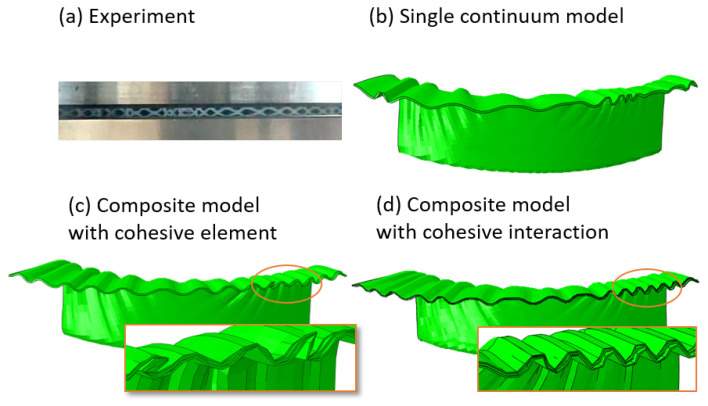
Comparison of wrinkle formation between experimental and simulation results of three material models (side view).

Figure 14 and Figure 15 illustrate the experimental and simulation results of three material models, i.e., (b) single continuum model, (c) multilayer composite model with cohesive element, and (d) multilayer composite model with cohesive interaction. First of all, all three material models simulate wrinkles, and the distribution of wrinkles is consistent with the experiment, which means that there are significantly more wrinkles distributed in the MD direction. Although the difference between the results of the three material models may not seem significant at first glance, it is noticeable that the composite model simulates a higher number of wrinkles and is also more similar to the actual distribution. This is particularly the case for the composite model with cohesive interaction, which has the most number of wrinkles (15, in contrast to 13 for the other two models). And the side view (ref. Figure 15) allows for a better distinction between the three material models, especially the two multilayer composite models. In deep drawing, the wrinkled area is located between the blank holder and the drawing die, and it is subjected to pressure in the thickness direction, as can be seen in Figure 15c, where the model undergoes element penetration in the simulation, while Figure 15d does not have this phenomenon. However, it is worth noting that the dual multilayer composite models required 2.5 times more time to compute than the single continuum model. Also, the experiment was performed at room temperature, and the simulation did not take into account thermal effects.

**Figure 16:**
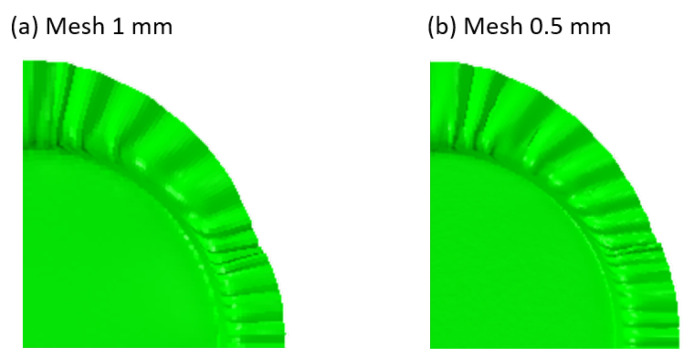
Comparison of the influence of mesh size on the wrinkle simulation.

It is important to note that the number of wrinkles in the simulation was significantly smaller than in the actual experiment. Additionally, the wrinkles appeared to be smoother than in the actual situation. The primary reason for this discrepancy is the insufficient size of the meshing. Another potential reason is the inhomogeneity of the material. Consequently, a continuous model with a mesh of 0.5 mm was employed in the simulation, which yielded a result of 68 wrinkles, an increase from the original 52 (ref. Figure 16). It is evident that the mesh division has a significant impact on the simulation of wrinkles. However, a finer mesh will inevitably result in a significant increase in computational time.

**Figure 17:**
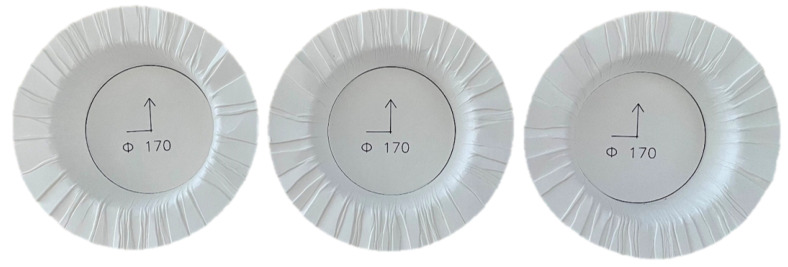
Comparison of three samples of deep drawing process under the same process parameter.

Due to the inhomogeneity of the paper and the complex friction conditions in a real experimental environment, it is not possible to find two identical specimens in the deep-drawing experiments, even though the process parameters are the same (ref. Figure 17). However, the results of the simulation will always be the same when the boundary conditions are the same. Therefore, it is not possible to obtain the same distribution in the simulation as in the experiment. Nevertheless, the comparison is sufficient to demonstrate the advantage of the composite models in the simulation of wrinkles. Since wrinkle formation is related to pressure in the thickness direction of the paperboard, the composite model with cohesive interactions is optimal in comparison, which can be reasonably explained.

## 4. Discussion

Three material models are proposed for paperboard material: a single continuum model, a composite model with cohesive elements, and a composite model with cohesive interaction. Various tests were performed to obtain the mechanical properties of the materials and to confirm the material models. The results indicated that uniaxial tensile tests in three directions (MD, 45°, and CD) are sufficient to determine the properties essential for in-plane modeling.

The material model for orthotropic elasticity and anisotropic plasticity is represented by a single continuum model. The model uses determined properties for the entire material and effectively captures in-plane behavior. It can also capture the wrinkling in the simulation of deep drawing to some extent. Linvill et al. [23] employed a perfectly plastic hinge model to enable the explicit finite element simulation of the entire deep-drawing process, taking into account wrinkle initiation. However, the approach of this article introduces certain assumptions and necessitates the addition of two additional material constants: one to model wrinkling in both the MD and CD. Additionally, the model has certain limitations in representing the material behavior in the thickness direction, as it does not consider delamination in the ZD.

The tensile test conducted out-of-plane revealed that delamination along the interface is the primary factor contributing to the inelastic behavior of the material in the ZD. Therefore, a cohesive interface is proposed and included in the composite material model along with the continuum model of single layers. The composite model can capture both in-plane and out-of-plane tensile behaviors. However, the infiltration of the cohesive elements also leads to compression deviation in the ZD. A three-layer model with cohesive interactions is established to simulate the effect of the cohesive interface on material behavior during the forming process. Cohesive contact provides cohesive bonding without penetration under pressure but results in a longer computational time, compared to cohesive elements. The model consisting of the layer models, and the cohesive contact interaction best captures the material behavior, as shown by the comparison between the FEM investigation and the experiment.

In conclusion, all three models have practical applications. The single continuum model is suitable for processes where delamination does not play a significant role, such as deep drawing and press forming, where the main concern is in-plane deformations. The composite model with cohesive elements is appropriate for procedures that involve delamination, such as converting. Lastly, the composite model with cohesive interaction is useful for processes that involve delamination with compressive loads, such as creasing. The composite model may yield better results when considering specific needs, such as simulating wrinkles during deep drawing. For a more detailed comparison and application, see Table 4.

## 5. Conclusions

Paperboard materials are becoming increasingly popular in the packaging industry as a sustainable alternative to plastics due to their recyclability. This study aims to characterize and model the delamination behavior of paperboard through experimental and numerical investigations. In-plane and out-of-plane shear tests are developed to validate empirical formulas and obtain parameters describing the out-of-plane shear behavior. Three material models, including the single continuum model, the delamination model with cohesive elements, and the delamination model with cohesive interaction, are developed for two different three-layer paperboards. Incorporating cohesive behavior between the layers is a suitable way to simulate the delamination behavior of paperboard in 2D forming and wrinkling in 3D forming as well. Considering the advantages and disadvantages of the two methods of generating the cohesive interface, it is crucial to choose the appropriate material model for a specific application scenario, i.e., when the compressive stress in the thickness direction is high, cohesive interaction should be chosen instead of cohesive element.The single continuum model, despite the need for less qualitative experiments and higher computational efficiency, is not able to represent the delamination phenomenon.

## Figures and Tables

**Table 1:** Material constant values of whole paperboard and individual layers (in-plane).

Parameter [Unit]	$E_{x}$ [MPa]	$E_{y}$ [MPa]	$E_{z}$ [MPa]	$G_{\mathrm{xy}}$ [MPa]	$\sigma_{0}$ [MPa]	a	b	c	d	$R_{\mathrm{xx}}$	$R_{\mathrm{xy}}$
Continuum	7200	2750	24	1806	3.7	−29.04	34.47	233.28	2.13	2.33	0.82
Layer1	7100	2300	24	2421	5.4	−93.55	21.88	688.79	1.56	2.52	0.97
Layer2	5450	1650	24	1972	7.3	−146.60	11.05	687.37	1.45	2.36	1
Layer3	6650	2300	24	1744	6.5	−31.84	24.70	271.53	1.90	2.22	0.9

**Table 2:** Material constant values of cohesive element and interaction (in Abaqus as an example).

Parameter [Unit]	$K_{\mathrm{nn}}$ [MPa/mm]	$K_{\mathrm{ss}}$ [MPa/mm]	$K_{\mathrm{tt}}$ [MPa/mm]	$t_{n}^{0}$ [MPa]	$t_{s}^{0}$ [MPa]	$t_{t}^{0}$ [MPa]	$\delta_{m}^{\max}$ [mm]	α [-]
Constants	25	0.075	0.075	0.34	0.12	0.12	5.2	11

**Table 3:** Geometric parameters and boundary conditions used in deep drawing simulations.

Parameter [Unit]	Geometric Parameter				Process Parameter	
**Components**	**Paperboard**	**Punch**	**Blank Holder**	**Die**		
Distance to centerline [mm]	0	0	41	40	Friction coefficient [-]	0.08
Height [mm]	0.42	58	50	50	Blank holder force [N]	1250
Width/Radius [mm]	65	39.55	49	80	Drawing depth [mm]	15
Chamfer diameter [mm]	-	R 0.2	0	R 3		

**Table 4:** Comparison of the three models in terms of advantages and disadvantages and their application.

Model	Advantages	Disadvantages	Application
Single continuum mode	- Simpler material characterization due to fewer material parameters required, i.e., fewer characterization experiments needed; - Higher computational efficiency	- Delamination failure in ZD cannot be considered; - Influence of delamination on wrinkle formation cannot be mapped	Processes without delamination, mainly in-plane deformation, such as 3D forming processes (deep drawing, press forming, and hydroforming) and embossing
Composite model with cohesive elements	- Material model accounts for delamination and failure in ZD; - Equally accurate description of in-plane deformation	- Complex material characterization due to more material parameters required; - Elements penetrate under a high compressive stress in ZD;	Process with delamination, no apparent compressive stress in thickness direction, like folding and opening process of packaging
Composite model with cohesive interface	- Material model accounts for delamination, failure, as well as under compressive stress in ZD; - Equally accurate description of in-plane deformation	- Complex material characterization due to more material parameters required; - Requires longer computation time	Process involving delamination with apparent compressive stress in the thickness direction, such as creasing and wrinkling in deep drawing

## Data Availability

The original contributions presented in the study are included in the article, further inquiries can be directed to the corresponding authors.
